# Application of the Healthy Eating Index-2015 and the Nutrient-Rich Food Index 9.3 for assessing overall diet quality in the Japanese context: Different nutritional concerns from the US

**DOI:** 10.1371/journal.pone.0228318

**Published:** 2020-01-30

**Authors:** Kentaro Murakami, M. Barbara E. Livingstone, Aya Fujiwara, Satoshi Sasaki

**Affiliations:** 1 Department of Social and Preventive Epidemiology, School of Public Health, University of Tokyo, Tokyo, Japan; 2 Nutrition Innovation Centre for Food and Health (NICHE), School of Biomedical Sciences, Ulster University, Coleraine, United Kingdom; University Sains Malaysia, MALAYSIA

## Abstract

**Objectives:**

While it is widely perceived that the diet consumed by Japanese is healthy, empirical evidence supporting this notion is limited. In this cross-sectional study, we assessed the overall diet quality of Japanese using the Healthy Eating Index-2015 (HEI-2015) and Nutrient-Rich Food Index 9.3 (NRF9.3), and compared diet quality scores between Japanese and Americans.

**Methods:**

We used 1-d dietary record data from 19,719 adults (aged ≥20 y) in the Japanese National Health and Nutrition Survey 2012 and the first 24-h dietary recall data from 4614 adults in the US NHANES 2011–2012.

**Results:**

As expected, a higher total score of the HEI-2015 and NRF9.3 was associated with favorable patterns of overall diet in the Japanese population. The range of total score was wide enough for both HEI-2015 (5th percentile 37.2; 95th percentile 67.2) and NRF9.3 (5th percentile 257; 95th percentile 645). Both HEI-2015 and NRF9.3 distinguished known differences in diet quality between sex, age, and smoking status. The mean total scores of HEI-2015 and NRF9.3 were similar between Japanese (51.9 and 448, respectively) and US adults (52.8 and 435, respectively). However, component scores between the 2 populations were considerably different. For HEI-2015, Japanese had higher scores for whole fruits, total vegetables, green and beans, total protein foods, seafood and plant proteins, fatty acids, added sugars, and saturated fats, but lower scores for total fruits, whole grains, dairy, refined grains, and sodium. For NRF9.3, the intakes of vitamin C, vitamin D, potassium, added sugars, and saturated fats were more favorable in Japanese, while those of dietary fiber, vitamin A, calcium, iron, magnesium, and sodium were less favorable.

**Conclusions:**

This study suggests the usefulness of HEI-2015 and NRF9.3 for assessing the diet quality of Japanese, as well as for highlighting different nutritional concerns between Japan and the US.

## Introduction

Partly due to the low prevalence of coronary artery disease and long life expectancy of the Japanese population, it is widely perceived that the diet consumed by Japanese is healthy [1–3]. However, empirical evidence supporting this assertion is limited, mainly because of a lack of appropriate assessment tools for the overall quality of the Japanese diet. For example, a number of studies have consistently shown that compliance with Japanese healthy eating guidelines (Japanese Food Guide Spinning Top) was associated with not only favorable aspects of dietary intake (e.g., higher intakes of dietary fiber and micronutrients) but also with unfavorable aspects (e.g., higher intakes of saturated fats and sodium) [4–7]. On the other hand, 2 large-scale prospective cohort studies have shown inverse associations between compliance and total mortality, among women at least [4,8]. Further, several studies suggest that both the Mediterranean diet score and the Dietary Approach to Stop Hypertension score may not be suitable for assessing the quality of the Japanese diet [7,9,10], partly due to a lack of important components (e.g., sodium for the former and refined grains for the latter). More importantly, these diet quality measures cannot be used to compare diet quality between different populations (e.g., Japanese and Americans), because compliance with the Japanese food guide is specific for the Japanese diet while the scoring system of both the Mediterranean diet score and Dietary Approach to Stop Hypertension score depends on the distribution of the population examined.

We recently conducted a systematic review of Japanese studies which derived dietary patterns from principal component analysis, and found that the food groups contributing to so-called healthy dietary patterns (fruits, vegetables, potatoes, mushrooms, seaweeds, and pulses) are at least partly similar to those commonly observed in Western countries (fruits, vegetables including mushrooms, poultry, fish, low-fat dairy, legumes, and whole grains) [11]. This finding indicates that a diet quality measure which covers a wide spectrum of dietary intake may be used to assess the quality of the Japanese diet even when the measure was developed for the Western dietary context. We speculated that the Healthy Eating Index-2015 (HEI-2015) [12–14] is a promising tool in this respect because, being based mainly on foods, it is designed to assess adherence to the latest and highly comprehensive dietary guidelines (for Americans) [15] and uses a scoring system which is independent of the distribution of the studied population. The Nutrient-Rich Food Index 9.3 (NRF9.3) [16–19] shares these characteristics, but is based on nutrient rather than food intake. To our knowledge, however, no attempt has yet been made to assess the feasibility and utility of these diet quality measures, initially developed in Western countries, in a non-Western dietary context, including Japan.

Consequently, the aims of this cross-sectional study were to assess the overall quality of the Japanese diet using HEI-2015 and NRF9.3, and to compare diet quality scores between Japanese and Americans using data from national dietary surveys, namely the Japanese National Health and Nutrition Survey (NHNS) and the US National Health and Nutrition Examination Survey (NHANES).

## Methods

### Japan dataset (NHNS)

The NHNS is a national nutrition survey conducted annually since 1945 by public health centers under the supervision of the Ministry of Health, Labour and Welfare, and in accordance with the Health Promotion Law. The present cross-sectional analysis used data from the 2012 NHNS, with permission. The 2012 survey was selected because it had a larger sample size than more recently available surveys. The NHNS has been detailed elsewhere [20,21]. Briefly, on the basis of the population census, 475 of the approximately one million census units were sampled randomly as survey areas. All non-institutionalized Japanese aged ≥1 y living in a survey area (approximately *n* = 61,000) were asked to participate, excluding individuals holding foreign citizenship, those whose diet was not self-selected, and those following a special diet (mainly due to disease). The survey was conducted between 25 October and 7 December 2012.

A total of 12,750 of 24,555 eligible households (52%) participated in NHNS 2012. The number of participants aged ≥20 y was 30,639. Of these, the number with missing information on dietary intake, anthropometric measurements, and lifestyle variables was 3913, 8593, and 473, respectively (some had more than one missing variable), resulting in 20,099 participants with complete information. After further excluding 246 lactating and 136 pregnant women, the final sample in this analysis comprised 19,717 male participants and non-lactating and non-pregnant female participants aged ≥20 y (S1 Fig). These participants differed somewhat from those excluded from the analysis, who were more likely to be male, younger, a current smoker, and to have lower mean energy intake and BMI (all *P* <0.0001).

This survey was conducted according to the guidelines laid down in the Declaration of Helsinki, and verbal informed consent was obtained from all individual participants. Under the Statistics Act, the Ministry of Health, Labour and Welfare anonymized individual-level data collected from the NHNS, and provided the first author with the datasets for this study. In accordance with the Ethical Guidelines of Epidemiological Research established by the Ministry of Education, Culture, Sports, Science and Technology and the Ministry of Health, Labour and Welfare, institutional review board approval was not required for this analysis.

### Dietary assessment

Dietary intake data were obtained using a 1-d weighed household dietary record, as detailed previously [21,22–24]. The dietary record therefore included data on all members of the household. In brief, the main record-keeper was supplied with a diary for recording and given written and verbal instructions in the home from trained fieldworkers (registered dietitians) on how the diary should be maintained. The main record-keeper was asked to weigh and record all food and beverage items (except drinking water) consumed by the household members during the day of recording. The equipment used to weigh foods and beverages was not provided, mainly due to funding limitations, but is typically found in most Japanese households. Where household members shared food items from the same dish, the record-keeper was requested to record the approximate proportions of food taken by each member to allow the dietary intake of each individual to be estimated. Where weighing was not possible (e.g., eating out), the record-keeper was asked to record as much detail as possible, including portion size and leftovers. To ensure maximum participation, the recording day could be freely selected by the household from any day, excluding Sundays, national holidays, and days with special events, such as wedding parties and funerals. Although the survey did not formally collect information on the identity of the main record-keeper, the survey assumed that recording was undertaken by the main household cook, who in Japan is usually a woman. Within a short time after recording (usually the next weekday), trained fieldworkers went to each household to confirm the completeness of food recording and then collect the diary. Additional information was added where necessary.

In accordance with a study manual of the NHNS, trained fieldworkers converted the estimates of portion sizes into weights (for food items recorded using household measures), and coded all individual food items on the basis of the Standard Tables of Food Composition in Japan [25]. The collected dietary records were then checked at the local center, where trained fieldworkers input the dietary intake data using software custom-developed for the NHNS. These data were then compiled by trained investigators at the central office to produce the overall dietary dataset. Estimated daily intakes of foods, energy, and nutrients for individuals were calculated using the household food consumption record, and for shared dishes and foods, approximate proportions consumed by each household member, based on the Standard Tables of Food Composition in Japan [25]. Added sugar was estimated based on a recent comprehensive composition database [26]. Dietary supplements were not considered during the nutrient intake calculation because it was our intention to assess only nutrient intake from foods and beverages.

The validity of this household dietary record in estimating individual-level dietary intake in Japanese has been examined [27]. Briefly, dietary intakes among young women (age about 20 y) estimated using this 1-d household dietary record by mothers (mean age 49 y) were compared with those estimated using a 1-d weighed dietary record that was independently conducted by the young women themselves (*n* = 32). Mean intake differences between the methods were 6.2% for energy, 5.7% for protein, 6.7% for fat, and 6.3% for carbohydrate, while Pearson correlation coefficients were 0.90 for energy, 0.89 for protein, 0.91 for fat, and 0.90 for carbohydrate. Further, previous analyses using the NHNS 2012 showed mean values for the ratio of energy intake to estimated energy requirement of 1.04 for children [28] and 0.98 for adults [29].

### Calculation of HEI-2015

Overall diet quality was assessed using the HEI-2015, as described in detail elsewhere [12–14]. Briefly, HEI-2015 is a composite measure of compliance with the 2015–2020 Dietary Guidelines for Americans [15], which has been well validated in the US population [13,14]. HEI-2015 is a 100-point scale, with a higher score indicating a better quality of overall diet. The adequacy components (maximum score) are total fruits (5), whole fruits (5), total vegetables (5), greens and beans (5), whole grains (10), dairy (10), total protein foods (5), seafood and plant proteins (5), and fatty acids (ratio of the sum of polyunsaturated and monounsaturated fatty acids to saturated fatty acids, 10). The moderation components are refined grains (10), sodium (10), added sugars (10), and saturated fats (10).

Because the scoring system of HEI-2015 is based on cup or ounce equivalents for food components, which are available for US foods (in the Food Patterns Ingredients Database (FPID) [30] but not Japanese foods, a cup and ounce equivalent database for food items appearing in the Standard Tables of Food Composition in Japan [25] was prepared using the FPID before application of HEI-2015 to the NHNS dataset. First, 289 of 2229 food items were identified as those containing no food components related to HEI-2015 (e.g., sugars, vegetable oils, and seasonings), for which the assignment of cup or ounce equivalents was not needed. Second, ounce equivalents for meat and seafood items (*n* = 715) were determined by the FPID definition of those for protein foods (1 ounce of cooked lean meat, poultry, or seafood can have no more than 2.63 g of allowable fat per 28.35 g of lean meat). Third, for intact grains or grain products such as rice (*n* = 122), 28.35 g of grains was defined as equal to 1 ounce grain equivalent (in accordance with FPID). Fourth, for fruit juice drinks (*n* = 19), the cup equivalent was determined based on the concentration of pure fruit juice as well as its cup equivalent (in accordance with FPID). Fifth, cup or ounce equivalent values were assigned for foods with a direct match in FPID (mainly fruits, vegetables, eggs, beans, legumes, dairy; *n* = 843). When there was no direct match, cup or ounce equivalent values of closely related food items in FPID were assigned (mainly Japanese vegetables and Japanese confectioneries, *n* = 204). For example, pickled turnip was assigned the value of raw turnip, while wheat-based Japanese cracker was assigned the value of pretzels. Finally, for other food items such as konjac (*n* = 37), the cup or ounce equivalents were determined by the value of ingredients shown in FPID as well as the yield percentage.

HEI-2015 component and total scores were then calculated for each participant, based on energy-adjusted values (except for fatty acids), namely as cup or ounce equivalents (for food components) and amount (for sodium) per 1000 kcal of energy and percentage of energy (for added sugars and saturated fats).

### Calculation of NRF9.3

Overall diet quality was also assessed using the NRF9.3, as described in detail elsewhere [16–19]. In short, the NRF9.3 is a validated, composite measure of the nutrient density of the total diet, calculated as the sum of the percentage of reference daily values (RDVs) for 9 qualifying nutrients minus the sum of the percentage of RDVs for 3 disqualifying nutrients. To allow a direct comparison of dietary quality between Japanese and Americans, reference daily values based on the US Food and Drug Administration and other standards in the US were used [19]. The 9 qualifying nutrients (and RDVs) were as follows: protein (50 g), dietary fiber (28 g), vitamin A (900 μg retinol activity equivalent), vitamin C (90 mg), vitamin D (20 μg), calcium (1300 mg), iron (18 mg), potassium (4700 mg), and magnesium (420 mg). The 3 disqualifying nutrients (and RDVs) were added sugars (50 g), saturated fats (20 g), and sodium (2300 mg). The daily intake of each nutrient for each participant was adjusted for energy intake (per 2000 kcal) and expressed as a percentage of the RDV. For qualifying nutrients, percentages of RDV were terminated at 100 such that a high intake of one nutrient would not compensate for the low intake of another. With regard to disqualifying nutrients, consideration was limited to the share which exceeded the recommended amount. Accordingly, a higher NRF9.3 total score indicates a better quality of the overall diet, and a maximum possible total score of 900 indicated a diet in which intakes per given amount of energy (2000 kcal) were above the RDVs for the 9 qualifying nutrients but below the RDVs for the 3 disqualifying nutrients.

### Assessment of basic characteristics

In the 2012 NHNS, anthropometric measurements were performed for 69% of the participants by trained fieldworkers using standardized procedures. Body height (nearest 0.1 cm) and weight (nearest 0.1 kg) were measured while the participants were barefoot and wearing light clothes only. Of the remaining 31% of participants, these 2 variables were measured by other household members at home or were self-reported. BMI (kg/m^2^) was calculated by the commonly used formula, namely weight (kg) divided by height squared (m^2^). Weight status in adults was defined based on BMI according to WHO recommendations [31], namely underweight <18.5, normal weight ≥18.5 to <25, overweight ≥25 to <30, and obese ≥30. Information on occupation (professional/manager, sales/service/clerical, security/transportation/labor, or not in paid employment) and current smoking (no or yes) was obtained using a self-administered questionnaire.

### US dataset (NHANES)

To compare overall diet quality between Japan and the US, we used data from NHANES 2011–2012, which is a national representative survey of the non-institutionalized civilian US population [32,33]. The documentation and data for NHANES were downloaded from the NHANES website [34]. The unweighted response rate for persons examined in NHANES 2011–2012 was 70% [35]. NHANES was conducted according to the guidelines laid down in the Declaration of Helsinki, and all procedures involving human subjects were approved by the National Center for Health Statistics Research Ethics Review Board. Written informed consent was obtained from each participant.

The analytic sample was limited to adults aged ≥20 y with complete and reliable, self-reported, 24-h dietary recall data for the first day (*n* = 4752). After excluding pregnant (*n* = 57) and lactating (*n* = 23) participants, as well as those with missing information on the variables of interest (*n* = 58), the final analytic sample included 4614 participants (S2 Fig). Dietary assessment in NHANES was done using an automated 5-step multiple pass approach, namely the USDA Automated Multiple-Pass Method [34,36–39]. Data collection was conducted on all days of the week throughout the year, although the number of days of the week was not necessarily proportional and seasonality was not accounted for. The component and total scores of HEI-2015 and NRF9.3 were calculated as described above, based on public domain data on the first day’s total nutrient intake (obtained from the NHANES website) [34] and Food Patterns Equivalents for foods (obtained from USDA Food Patterns Equivalents Database website) [40].

### Statistical analysis

All statistical analyses were performed using SAS statistical software (version 9.4, SAS Institute Inc, Cary, North Carolina). Data are presented as means ± SDs for continuous variables and as the numbers and percentages of participants for categorical variables. Using the Japanese data, we first examined intakes of nutrients and food groups according to tertile category of HEI-2015 and NRF9.3 to examine if these diet quality measures (originally developed in Western countries) are actually useful in assessing overall diet quality among Japanese. For this, general linear models were used with adjustment for age, sex, weight status, occupation, and current smoking. Second, we examined the distributions of the total and component scores of HEI-2015 and NRF9.3 to assess if the distribution was wide enough to detect meaningful differences. Third, we calculated Pearson correlation coefficients among the total and component scores of each of HEI-2015 and NRF9.3 to examine if the respective components could capture a different aspect of dietary intake. Additionally, we calculated Pearson correlation coefficients between energy intake and the total and component scores of HEI-2015 and NRF9.3 if these diet quality measures were found to be able to assess diet quality independent of diet quantity, as measured by the energy value of the diet. Fourth, we examined whether HEI-2015 and NRF9.3 could distinguish between groups with known differences in the quality of their diets. Given previous findings that men have poorer quality diets than women, young adults have poorer quality diets than older adults, and current smokers have poorer quality diets than nonsmokers [13,14,19], we assessed the ability of HEI-2015 and NRF9.3 to distinguish differences in diet quality using an independent *t*-test. Finally, we compared the total and component scores of HEI-2015 and NRF9.3 between Japanese and US adults, using general linear models with adjustment for age, sex, and current smoking.

Data have not been weighted to take account of unequal probabilities of selection resulting from the sample design and non-response, because of a lack of information for doing so (i.e., sampling weights) in NHNS [20,21]. Because of the large sample size and number of statistical tests performed, significance was set for 2-tailed tests at *P* <0.001.

## Results

### Characteristics of the analytic sample

This analysis included 19,717 Japanese adults (aged ≥20 y) from NHNS 2012 and 4614 US adults from NHANES 2011–2012 (Table 1). There were more women in the Japanese dataset than in the US dataset. The Japanese sample had a higher mean age and a lower mean height, weight, BMI, and energy intake than the US sample, whereas the prevalence of current smokers was similar between the 2 countries.

**Table 1:** Basic characteristics of participants aged ≥20 y in the Japanese National Health and Nutrition Survey 2012 (*n* = 19717) and US NHANES 2011–2012 (*n* = 4614)^1^.

	Japan	US
Sex, *n* (%)		
Male	8712 (44.2)	2334 (50.6)
Female	11005 (55.8)	2280 (49.4)
Age, y	57.5 ± 16.7	48.5 ± 17.6
Body height, m	1.59 ± 0.10	1.68 ± 0.10
Body weight, kg	58.7 ± 11.8	81.5 ± 21.3
BMI, kg/m^2^	23.0 ± 3.5	28.9 ± 6.9
Weight status, *n* (%)		
Underweight (BMI <18.5)	1498 (7.6)	83 (1.8)
Normal weight (BMI ≥18.5 to <25)	13250 (67.2)	1355 (29.4)
Overweight (BMI ≥25 to <30)	4217 (21.4)	1478 (32.0)
Obese (BMI ≥30)	752 (3.8)	1698 (36.8)
Occupation, *n* (%)		
Professional/manager	2745 (13.9)	—
Sales/service/clerical	4838 (24.5)	—
Security/transportation/labor	3844 (19.5)	—
Not in paid employment	8290 (42.0)	—
Current smoking, *n* (%)		
No	16135 (81.8)	3658 (79.3)
Yes	3582 (18.2)	956 (20.7)
Energy intake, kcal/d	1938 ± 565	2152 ± 1005

^1^ Values are means ± SDs unless otherwise indicated.

### Application of HEI-2015 and NRF9.3 to the Japanese NHNS dataset

As expected, a higher total score of HEI-2015 and NRF9.3 was associated with favorable patterns of overall diet in the Japanese population (S1 Table for nutrients and S2 Table for food groups), suggesting the usefulness of these 2 diet quality measures for assessing overall diet quality in Japanese. The mean total score of the HEI-2015 was 52.2 (maximum score 100), while that of the NRF9.3 was 452 (maximum score 900) (Table 2). The Pearson correlation coefficient between the total score of HEI-2015 and that of NRF9.3 was 0.66.

**Table 2:** Mean values and percentiles of component and total scores of HEI-2015 and NRF9.3 among participants aged ≥20 y in the Japanese National Health and Nutrition Survey 2012 (*n* = 19717)^1^.

						Percentile				
	Mean ± SD	1st	5th	10th	25th	50th	75th	90th	95th	99th
HEI-2015^2^										
Adequacy components										
Total fruits (5)	2.0 ± 2.0	0.0	0.0	0.0	0.0	1.7	4.0	5.0	5.0	5.0
Whole fruits (5)	2.6 ± 2.3	0.0	0.0	0.0	0.0	3.2	5.0	5.0	5.0	5.0
Total vegetables (5)	4.6 ± 0.9	0.9	2.3	3.3	5.0	5.0	5.0	5.0	5.0	5.0
Greens and beans (5)	2.5 ± 2.2	0.0	0.0	0.0	0.0	2.6	5.0	5.0	5.0	5.0
Whole grains (10)	0.5 ± 1.9	0.0	0.0	0.0	0.0	0.0	0.0	0.0	4.1	10.0
Dairy (10)	1.8 ± 2.2	0.0	0.0	0.0	0.0	0.9	3.0	4.9	6.1	9.0
Total protein foods (5)	4.6 ± 0.8	1.3	2.6	3.4	4.7	5.0	5.0	5.0	5.0	5.0
Seafood and plant proteins (5)	4.4 ± 1.4	0.0	0.1	1.8	5.0	5.0	5.0	5.0	5.0	5.0
Fatty acids (10)	6.9 ± 3.1	0.0	0.9	2.2	4.5	7.5	10.0	10.0	10.0	10.0
Moderation components										
Refined grains (10)	1.6 ± 2.6	0.0	0.0	0.0	0.0	0.0	2.6	5.7	7.7	10.0
Sodium (10)	2.0 ± 3.0	0.0	0.0	0.0	0.0	0.0	3.5	7.1	9.1	10.0
Added sugars (10)	9.5 ± 1.2	4.1	6.9	8.1	9.8	10.0	10.0	10.0	10.0	10.0
Saturated fats (10)	9.2 ± 1.7	1.7	5.2	6.8	9.2	10.0	10.0	10.0	10.0	10.0
Total score^3^	52.2 ± 9.2	30.3	37.2	40.8	46.2	52.2	58.2	63.5	67.2	75.2
NRF9.3										
Protein	100 ± 3	87	100	100	100	100	100	100	100	100
Dietary fiber	57 ± 20	19	27	32	42	54	70	86	97	100
Vitamin A	55 ± 27	6	15	20	33	52	76	100	100	100
Vitamin C	88 ± 22	16	38	52	84	100	100	100	100	100
Vitamin D	38 ± 33	1	3	5	10	24	62	100	100	100
Calcium	41 ± 18	12	17	20	27	38	52	66	77	99
Iron	48 ± 15	21	27	31	37	46	57	68	77	99
Potassium	60 ± 18	26	34	39	47	59	72	86	95	100
Magnesium	67 ± 17	33	41	46	54	66	79	93	100	100
Added sugars	4 ± 16	0	0	0	0	0	0	2	25	81
Saturated fats	5 ± 13	0	0	0	0	0	0	17	31	62
Sodium	94 ± 67	0	3	18	47	84	129	182	218	299
Total score^4^	452 ± 118	166	257	303	375	452	531	604	645	721

^1^ A higher score indicates a higher diet quality, except for added sugars, saturated fats, and sodium components in NRF9.3, for which a higher score indicates an unfavorable dietary intake (i.e., higher intakes of added sugars, saturated fats, and sodium). HEI, Healthy Eating Index; NRF9.3, Nutrient-Rich Food Index 9.3.

^2^ Maximum score for each component indicated in parentheses. For moderation components, higher scores reflect lower intake.

^3^ Calculated as the sum of all component scores.

^4^ Calculated as the sum of scores for nine nutrients to encourage (i.e., protein, dietary fiber, vitamins A, C, and D, calcium, iron, potassium, and magnesium) minus the sum of scores for three nutrients to limit (i.e., added sugar, saturated fats, and sodium).

The range of total score was wide enough to allow meaningful differences to be detected for both HEI-2015 (from 30.3 at the 1st to 75.2 at the 99th percentiles) and NRF9.3 (from 166 at the 1st to 721 at the 99th percentiles) (Table 2). For almost all components of HEI-2015, scores at the 1st percentile were near 0, while those at the 99th percentile were at their optimum value. The added sugar component was the only one for which even the bottom 1% of the population achieved a relatively high score (4.1 of 10). At the 5th percentile, scores were near 0 for most components, except those representing total vegetables, total protein foods, added sugars, and saturated fats. At the 95th percentile, scores were at or near the maximum score for most components, except those representing whole grains, dairy, and refined grains. Additionally, more than half of participants achieved the minimum score for whole grains, refined grains, and sodium, while more than half of them achieved the maximum score for total vegetables, total protein foods, seafood and plant proteins, added sugars, and saturated fats. In terms of the components of NRF9.3, the range of component scores was also wide enough, despite the fact that more than half of these participants had maximum scores for protein, vitamin C, added sugars, and saturated fats.

For HEI-2015, the correlations among component scores were generally low (absolute values ≤0.25), except for those between overlapping components (0.92 between total fruits and whole fruits, 0.28 between total vegetables and greens and beans, 0.54 between total protein foods and seafood and plant proteins, and 0.47 between fatty acids and saturated fats) as well as those between whole and refined grains (0.36) and between dairy and fatty acids (-0.46). The correlations of the total with component scores were all positive (0.12 to 0.60), while those of the total and component scores with energy intake were sufficiently weak (-0.06 to 0.12). For NRF9.3, the correlations among component scores were generally low to moderate (absolute values ≤0.62), except for those between dietary fiber and potassium (0.79), dietary fiber and magnesium (0.70), iron and magnesium (0.75), and potassium and magnesium (0.81). The correlations of the total and component scores were all in the expected direction (0.18 to 0.71 for qualifying nutrients and ≤-0.16 for disqualifying nutrients), while those of the total and component scores with energy intake were sufficiently weak (absolute values ≤0.21).

The mean HEI-2015 total score for men was significantly lower than for women, and 6 of the 13 component scores were lower for men than for women (Table 3). The young age group (20–54 y) had a significantly lower mean total HEI-2015 score and lower scores for 10 of the 13 components compared with the older age group (≥55 y). Mean HEI-2015 total score for current smokers was significantly lower that for nonsmokers, and 7 of the 13 component scores were lower for smokers than for nonsmokers. Closely similar patterns were also observed for NRF9.3.

**Table 3:** Mean values of component and total scores of HEI-2015 and NRF9.3 according to sex, age group, and smoking status among participants aged ≥20 y in the Japanese National Health and Nutrition Survey 2012 (*n* = 19717)^1^.

	Male (*n* = 8712)	Female (*n* = 11055)	Age 20–54 y (*n* = 7847)	Age ≥55 y (*n* = 11870)	Nonsmoker (*n* = 16135)	Current smoker (*n* = 3582)
HEI-2015^2^						
Adequacy components						
Total fruits (5)	1.6 ± 1.9 ^3^	2.4 ± 2.0**	1.2 ± 1.7	2.6 ± 2.0**	2.2 ± 2.0	1.2 ± 1.7**
Whole fruits (5)	2.2 ± 2.2	3.0 ±2.2**	1.7 ± 2.1	3.3 ± 2.2**	2.9 ± 2.2	1.6 ± 2.1**
Total vegetables (5)	4.5 ± 1.0	4.7 ± 0.8**	4.5 ± 1.0	4.7 ± 0.8**	4.7 ± 0.8	4.3 ± 1.2**
Greens and beans (5)	2.3 ± 2.2	2.7 ± 2.2**	2.2 ± 2.2	2.8 ± 2.2**	2.7 ± 2.2	2.0 ± 2.2**
Whole grains (10)	0.5 ± 2.0	0.5 ± 1.9	0.4 ± 1.9	0.5 ±2.0	0.5 ± 2.0	0.4 ± 1.9
Dairy (10)	1.4 ± 1.9	2.1 ± 2.3**	1.6 ± 2.1	1.9 ± 2.2**	1.9 ± 2.2	1.2 ± 1.9**
Total protein foods (5)	4.6 ± 0.8	4.6 ± 0.9	4.5 ± 0.9	4.6 ± 0.8**	4.6 ± 0.8	4.5 ± 0.9**
Seafood and plant proteins (5)	4.4 ± 1.4	4.4 ± 1.4	4.0 ± 1.7	4.6 ± 1.2**	4.4 ± 1.4	4.2 ± 1.6**
Fatty acids (10)	7.1 ± 3.0	6.7 ± 3.2**	6.6 ± 3.2	7.0 ± 3.1**	6.8 ± 3.1	7.1 ± 3.1**
Moderation components						
Refined grains (10)	1.5 ± 2.5	1.7 ± 2.7**	1.5 ± 2.5	1.7 ± 2.6**	1.7 ± 2.6	1.5 ± 2.6
Sodium (10)	2.3 ± 3.2	1.8 ± 2.9**	2.4 ± 3.2	1.7 ± 2.8**	1.9 ± 2.9	2.3 ± 3.2**
Added sugars (10)	9.6 ± 1.2	9.4 ± 1.3**	9.5 ± 1.3	9.5 ± 1.2	9.5 ± 1.2	9.5 ± 1.4
Saturated fats (10)	9.3 ± 1.5	9.0 ± 1.9**	8.8 ± 2.1	9.4 ± 1.4**	9.2 ± 1.7	9.1 ± 1.8
Total score^4^	51.3 ± 9.0	52.9 ± 9.2**	48.9 ± 8.9	54.4 ± 8.7**	52.9 ± 9.1	48.9 ± 8.9**
NRF9.3						
Protein	99.5 ± 3.1	99.7 ± 2.2**	99.5 ± 3.2	99.7 ± 2.2**	99.7 ± 2.3	99.3 ± 3.9**
Dietary fiber	51 ± 19	61 ± 20**	49 ± 18	62 ± 20**	59 ± 20	47 ± 18**
Vitamin A	50 ± 26	60 ± 27**	51 ± 26	58 ± 27**	57 ± 27	46 ± 26**
Vitamin C	84 ± 24	91 ± 19**	82 ± 25	92 ± 18**	90 ± 20	79 ± 27**
Vitamin D	37 ± 33	38 ± 34	31 ± 31	42 ± 34**	39 ± 33	34 ± 32**
Calcium	37 ± 17	45 ± 19**	37 ± 17	44 ± 19**	43 ± 18	34 ± 16**
Iron	45 ± 14	51 ± 16**	44 ± 14	51 ± 15**	49 ± 15	43 ± 14**
Potassium	56 ± 17	64 ± 18**	54 ± 16	65 ± 18**	62 ± 18	53 ± 16**
Magnesium	63 ± 17	70 ± 17**	61 ± 16	71 ± 17**	69 ± 17	61 ± 17**
Added sugars	3 ± 15	4 ± 16**	4 ± 18	3 ± 14**	3 ± 15	4 ± 19*
Saturated fats	3 ± 11	5 ± 14**	7 ± 16	3 ± 10**	5 ± 13	5 ± 13
Sodium	87 ± 64	100 ± 68**	84 ± 64	101 ± 68**	96 ± 67	88 ± 67**
Total score^5^	429 ± 116	471 ± 117**	412 ± 113	478 ± 115**	464 ± 116	398 ± 111**

^1^ A higher score indicates a higher diet quality, except for added sugars, saturated fats, and sodium components in NRF9.3 for which a higher score indicates an unfavorable dietary intake (i.e., higher intakes of added sugars, saturated fats, and sodium). Difference between sex, age group, and smoking status was examined by the independent t-test, and significant differences from the counterpart group are shown using asterisk: * *P* <0.001; ** *P* <0.0001. HEI, Healthy Eating Index; NRF9.3, Nutrient-Rich Food Index 9.3.

^2^ Maximum score for each component indicated in parentheses. For moderation components, higher scores reflect lower intake.

^3^ Means ± SDs (all such values).

^4^ Calculated as the sum of all component scores.

^5^ Calculated as the sum of scores for nine nutrients to encourage (i.e., protein, dietary fiber, vitamins A, C, and D, calcium, iron, potassium, and magnesium) minus the sum of scores for three nutrients to limit (i.e., added sugar, saturated fats, and sodium).

### Comparison of HEI-2015 and NRF9.3 between Japan and the US

**Fig 1:**
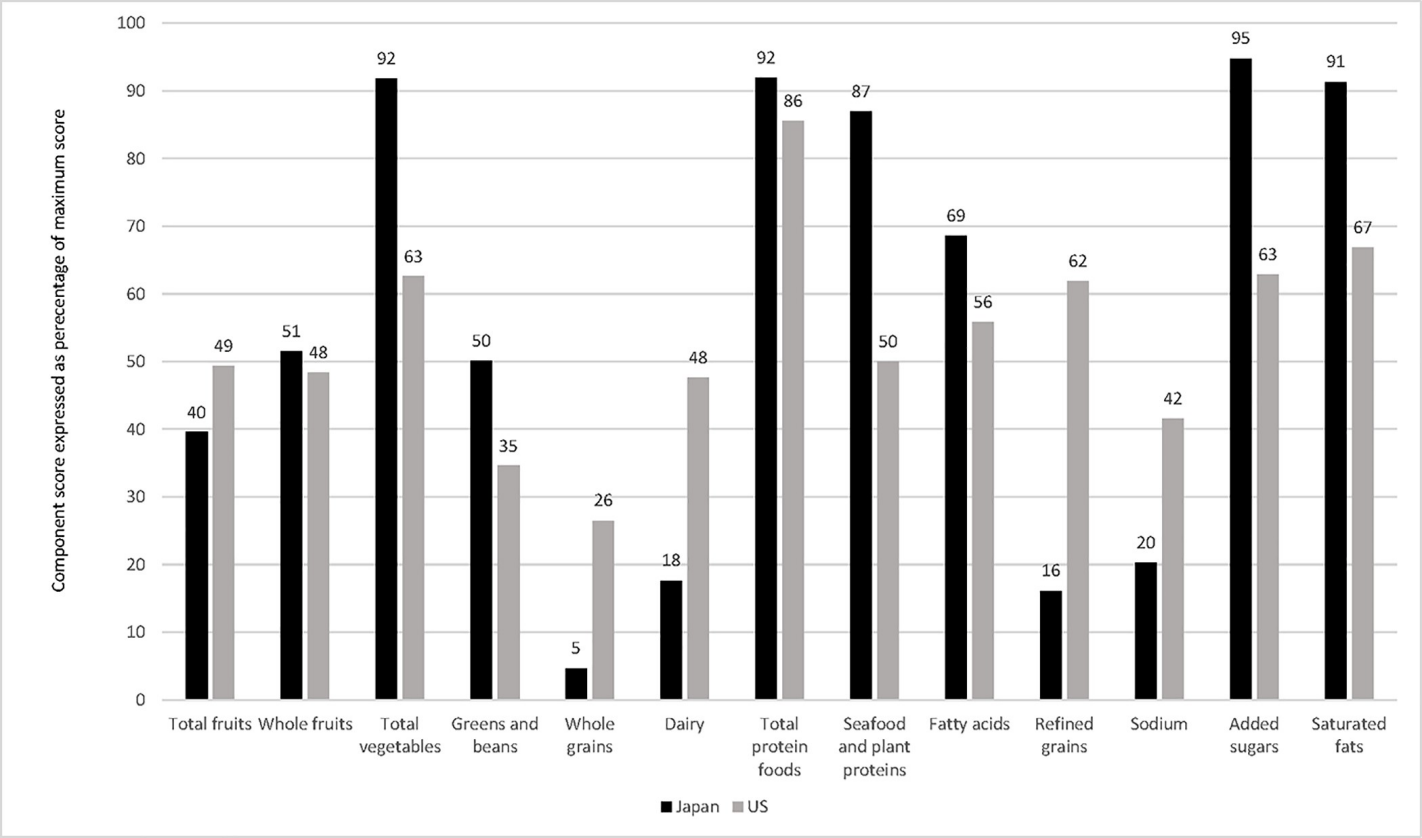
Comparison of the Healthy Eating Index-2015 (HEI-2015) component scores between participants aged ≥20 y in the Japanese National Health and Nutrition Survey 2012 (n = 19717) and those in the US NHANES 2011–2012 (n = 4614). Values are means adjusted for sex, age (y, continuous), and current smoking status (no or yes) and shown as a percentage of the maximum score. In HEI-2015, a higher score indicates a higher diet quality. For all components, the mean value was significantly different between the two countries (*P* <0.0001).

**Fig 2:**
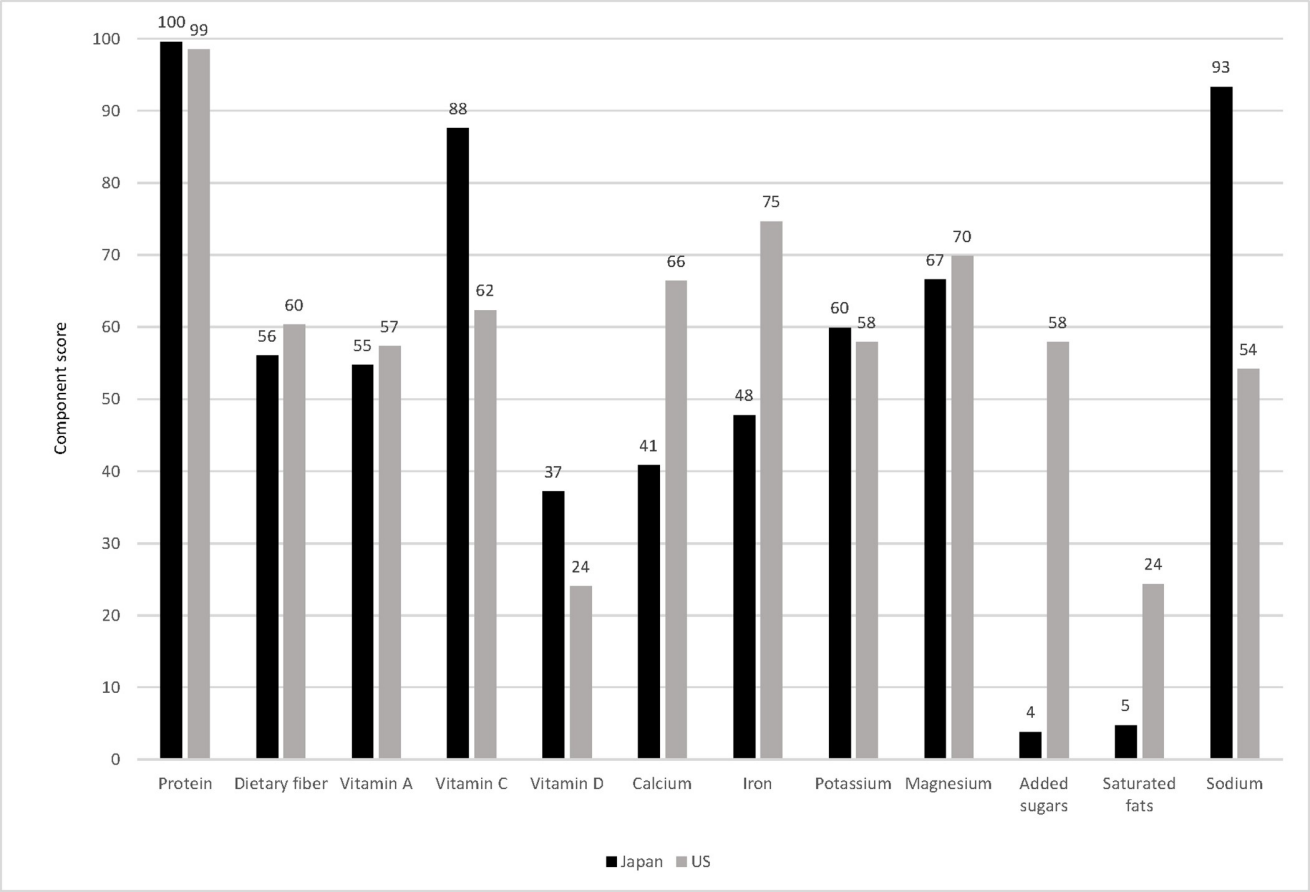
Comparison of the Nutrient-Rich Food Index 9.3 (NRF9.3) component scores between participants aged ≥20 y in the Japanese National Health and Nutrition Survey 2012 (*n* = 19717) and those in the US NHANES 2011–2012 (*n* = 4614). Values are means adjusted for sex, age (y, continuous), and current smoking status (no or yes). In NRF9.3, a higher score indicates a higher diet quality, except for added sugars, saturated fats, and sodium components, for which a higher score indicates an unfavorable dietary intake (i.e., higher intakes of added sugars, saturated fats, and sodium). For all components, the mean value was significantly different between the two countries (*P* <0.0001).

After adjustment for age, sex, and current smoking, the mean (± SE) total scores of HEI-2015 and NRF9.3 were similar between Japanese (51.9 ± 0.07 and 448 ± 0.9, respectively) and US adults (52.8 ± 0.15 and 435 ± 1.9, respectively), despite statistical significance (both *P* <0.0001) because of the large sample size. However, the component scores were considerably different between the 2 populations. For the HEI-2015 components, Japanese had not only higher mean scores of whole fruits, total vegetables, green and beans, total protein foods, seafood and plant proteins, fatty acids, added sugars, and saturated fats than the US counterpart, but also lower mean scores of other components, including total fruits, whole grains, dairy, refined grains, and sodium (Fig 1). Similar patterns were also found for the NRF9.3 components. The intakes of vitamin C, vitamin D, potassium, added sugars, and saturated fats were on average more favorable in Japanese than Americans, whereas those of other nutrients (including dietary fiber, vitamin A, calcium, iron, magnesium, and sodium) were less favorable (Fig 2).

## Discussion

In this study, we showed that a higher total score of the HEI-2015 and NRF9.3 was associated with favorable patterns of overall diet in the Japanese population. The range of total score was wide enough for both diet quality measures. Both HEI-2015 and NRF9.3 also distinguished known differences in diet quality between sex, age, and smoking status. While overall diet quality assessed as total scores of HEI-2015 and NRF9.3 was similar between Japanese and American adults, the component scores differed considerably between the 2 populations, suggesting different nutritional concerns between Japan and the US. To our knowledge, this study is the first to apply diet quality measures initially developed in Western countries to non-Western populations, and suggests the possible utility of these diet quality measures for international comparisons of dietary quality.

Recent Japanese diets typically include high intakes of refined grains (mainly white rice), seaweeds, vegetables, fish, soybean products, and green tea, as well as low intakes of whole grains, processed meat, nuts, and soft drinks [20,41]. This suggests that there are some similarities between typical Japanese and Western diets but they are still very different in terms of foods and thus nutrients as well as preparation techniques. Nevertheless, we found that both HEI-2015 and NRF9.3 were useful tools for assessing the diet quality of Japanese. This appears reasonable, not only because the food groups that contribute to healthy eating patterns appear to be generally similar between Japanese and Western countries [11], but also because nutrients of concern are generally similar in affluent societies [7,12,15,19]. It would be interesting to investigate whether similar findings are obtained for other Asian countries.

Interestingly, despite the popular notion that the Japanese diet is healthy [1–3], we found that overall diet quality as assessed by the HEI-2015 and NRF9.3 total scores was generally similar between Japanese and American adults. In contrast, the component scores differed considerably between the 2 populations. Compared with those for Americans, major concerns for Japanese included higher intakes of sodium and refined grains and lower intakes of dairy and whole grains. This is reasonably consistent with previous findings on the area of continuous concern of the Japanese diet, such as its high intake of salty foods (sodium) and high intake of refined grains (particularly white rice) [7,22,24]. It is also striking that considerably high scores were on average observed among five HEI-2015 subcomponents in the Japanese population (total vegetables, total protein foods, seafood and plant proteins, added sugars, and saturated fats). This may be related to the perception that the Japanese diet is healthy. In contrast, major concerns for Americans, compared with Japanese, were higher intakes of added sugars and saturated fats and lower intakes of total vegetables, green and beans, and seafood and plant proteins. This kind of finding, based as it is on a between-country comparison, would be useful in the broad identification of nutritional concerns, as well as in developing an appropriate strategy for improving diet quality within a country. Further cross-country investigation on diet quality should thus be facilitated.

Several limitations of this study are acknowledged. First, mainly because of the low response rate in the NHNS, we cannot rule out the possibility of selection bias, as discussed earlier in more detail [24]. The NHANES data shown here also would not be considered representative of the US population since weights were not used in this analysis to account for non-response as well as over-sampling of some demographic groups.

Second, it is not possible to completely avoid random and systematic errors in self-reported dietary assessment [28,42,43]. The validity of the NHNS’s household dietary record for estimating dietary intake at the individual level also remains a serious concern, as described earlier in more detail [24]. Because day-to-day variation in dietary intake exists in free-living settings, it is unlikely that dietary intakes derived from 1-d dietary records represent usual intake among individuals. This is particularly problematic for the calculation of HEI-2015, because at least several components of HEI-2015 are foods consumed episodically and thus 1-d dietary data are 0-inflated [44]. Thus, calculation of HEI-2015 based on only 1-d dietary data would result in a lower score than with the use of long-term dietary intake data or the estimation of usual distribution, as shown in previous studies in NHANES [13,45]. Further, dietary assessment in the NHNS was conducted without consideration of the day of the week as well as excluding Sundays, which may cause bias for estimating average dietary intake. Data on which day was selected for dietary assessment are not available [20]. Additionally, no consideration for possible seasonal variation in dietary intake was taken because of the limited survey period in the NHNS, which may also cause bias into the estimation of average dietary intakes [46]. Moreover, misreporting of dietary intake particularly among overweight and obese participants is a persistent problem [28,42,43]. Nevertheless, the calculation of both HEI-2015 and NRF9.3 was based on energy-adjusted dietary intake values, and the correlations between HEI-2015 and NRF9.3 total and component scores and energy intake was low.

Third, it should be noted that there are many methodological differences between the Japanese NHNS and US NHANES, particularly with regard to dietary assessment method (1-d household weighed dietary record vs a 24-h dietary recall) and the cup and ounce equivalent database (manually developed database for this study vs USDA Food Patterns Equivalents Database), in addition to individual foods that make up each component. The present results should thus be interpreted in this context. Finally, both HEI-2015 and NRF9.3 and their scoring systems were primarily developed to assess the diet quality of Americans. Thus, although many components that are emphasized in Japan (e.g., fish, vegetables, beans, dairy, fruits, rice, and sodium) [6,8,47,48] are also included in the HEI-2015, NRF9.3, or both, it should be stressed that these diet quality measures are not optimal for assessing he quality of Japanese diet, but rather the best available. A more convincing argument that the HEI-2015 and NRF9.3 can be used to assess diet in a Japanese population may be made by examining the association between these diet quality measures and clinical biomarkers and health outcomes, which is beyond the scope of this study. Further effort in developing country-specific food-based dietary guidelines (based on the present finding) is undoubtedly needed in Japan.

In conclusion, based on data from a national dietary survey, this study suggests the usefulness of both HEI-2015 and NRF9.3 in assessing the quality of diet consumed by Japanese adults. The findings may serve as both a reference and an indication for future research, as well as for the development of food-based dietary guidelines in Japan. When Japanese and American adults were compared, overall diet quality assessed as total scores of the HEI-2015 and NRF9.3 was similar. However, the component scores differed considerably between the 2 populations, suggesting different nutritional concerns between Japan and the US. This study also suggests the possible utility of these diet quality measures from a global perspective; their use in directly comparing diet quality between various countries would be interesting.

## Supporting information

S1 Fig Flow diagram of Japanese participants included in the present analysis. (DOCX)

S2 Fig Flow diagram of US participants included in the present analysis. (DOCX)

S1 Table Energy and nutrient intakes according to tertile (T) category of total scores of HEI-2015 and NRF9.3 among participants aged ≥20 y in the Japanese National Health and Nutrition Survey 2012 (*n* = 19717). (DOCX)

S2 Table Food group intakes (g/1000 kcal) according to tertile (T) category of total scores of HEI-2015 and NRF9.3 among participants aged ≥20 y in the Japanese National Health and Nutrition Survey 2012 (*n* = 19717). (DOCX)
