# Supplementary material for: Application of the Healthy Eating Index-2015 and the Nutrient-Rich Food Index 9.3 for assessing overall diet quality in the Japanese context: Different nutritional concerns from the US
Source: PLoS One. 2020 Jan 30;15(1):e0228318. doi: 10.1371/journal.pone.0228318 (PMC6992222; doi:10.1371/journal.pone.0228318)
Supplement: S1 Fig — (DOCX) [file pone.0228318.s001.docx]

S1 Fig. Flow diagram of Japanese participants included in the present analysis.
