# Supplementary material for: Application of the Healthy Eating Index-2015 and the Nutrient-Rich Food Index 9.3 for assessing overall diet quality in the Japanese context: Different nutritional concerns from the US
Source: PLoS One. 2020 Jan 30;15(1):e0228318. doi: 10.1371/journal.pone.0228318 (PMC6992222; doi:10.1371/journal.pone.0228318)
Supplement: S1 Table — (DOCX) [file pone.0228318.s003.docx]

S1 Table. Energy and nutrient intakes according to tertile (T) category of total scores of HEI-2015 and NRF9.3 among participants aged ≥20 y in the Japanese National Health and Nutrition Survey 2012 (*n* = 19717)^1^

|  |  | HEI-2015 |  |  |  | NRF9.3 |  |  |
| --- | --- | --- | --- | --- | --- | --- | --- | --- |
|  | T1 (median 43.6; *n* = 6572) | T2 (median 52.2; *n* = 6573) | T3 (median 60.6; *n* = 6572) | *P* for trend^2^ | T1 (median 341;  *n* = 6572) | T2 (median 452;  *n* = 6572) | T3 (median 565;  *n* = 6572) | *P* for trend^2^ |
| Energy (kcal/d) | 1878 ± 6 | 1933 ± 6 | 2002 ± 6 | <0.0001 | 1897 ± 7 | 1961 ± 6 | 1955 ± 7 | <0.0001 |
| Macronutrients |  |  |  |  |  |  |  |  |
| Protein (% energy) | 14.1 ± 0.04 | 15.0 ± 0.04 | 15.5 ± 0.04 | <0.0001 | 13.8 ± 0.04 | 14.7 ± 0.04 | 16.0 ± 0.04 | <0.0001 |
| Fat (% energy) | 25.8 ± 0.09 | 24.9 ± 0.09 | 25.7 ± 0.09 | 0.41 | 25.3 ± 0.09 | 25.5 ± 0.09 | 25.4 ± 0.09 | 0.30 |
| Saturated fats (% energy) | 7.8 ± 0.03 | 6.7 ± 0.03 | 6.5 ± 0.03 | <0.0001 | 7.2 ± 0.03 | 7.0 ± 0.03 | 6.8 ± 0.03 | <0.0001 |
| MUFA (% energy) | 9.3 ± 0.04 | 8.9 ± 0.04 | 9.2 ± 0.04 | 0.002 | 9.3 ± 0.04 | 9.2 ± 0.04 | 8.9 ± 0.04 | <0.0001 |
| n-6 PUFA (% energy) | 4.3 ± 0.02 | 4.7 ± 0.02 | 5.0 ± 0.02 | <0.0001 | 4.6 ± 0.02 | 4.7 ± 0.02 | 4.7 ± 0.02 | <0.0001 |
| n-3 PUFA (% energy) | 0.9 ± 0.01 | 1.2 ± 0.01 | 1.3 ± 0.01 | <0.0001 | 0.9 ± 0.01 | 1.1 ± 0.01 | 1.3 ± 0.01 | <0.0001 |
| Carbohydrate (% energy) | 56.5 ± 0.1 | 56.5 ± 0.1 | 55.5 ± 0.1 | <0.0001 | 56.2 ± 0.1 | 56.2 ± 0.1 | 56.1 ± 0.1 | 0.56 |
| Added sugars (% energy) | 5.4 ± 0.05 | 4.9 ± 0.05 | 4.8 ± 0.05 | <0.0001 | 5.5 ± 0.05 | 5.0 ± 0.05 | 4.6 ± 0.05 | <0.0001 |
| Dietary fiber (g/1000 kcal) | 6.8 ± 0.03 | 7.9 ± 0.03 | 9.4 ± 0.03 | <0.0001 | 6.4 ± 0.03 | 7.8 ± 0.03 | 10.0 ± 0.03 | <0.0001 |
| Micronutrients |  |  |  |  |  |  |  |  |
| Vitamin A (μg RAE/1000 kcal) | 241 ± 6 | 299 ± 6 | 352 ± 6 | <0.0001 | 174 ± 6 | 273 ± 5 | 446 ± 6 | <0.0001 |
| Vitamin D (μg/1000 kcal) | 3.4 ± 0.06 | 4.4 ± 0.06 | 5.3 ± 0.06 | <0.0001 | 2.7 ± 0.06 | 4.1 ± 0.06 | 6.3 ± 0.06 | <0.0001 |
| Vitamin E (mg/1000 kcal) | 3.1 ± 0.02 | 3.6 ± 0.02 | 4.3 ± 0.02 | <0.0001 | 3.0 ± 0.02 | 3.6 ± 0.02 | 4.4 ± 0.02 | <0.0001 |
| Vitamin K (μg/1000 kcal) | 98.5 ± 1.2 | 138.8 ± 1.2 | 171.1 ± 1.2 | <0.0001 | 90.2 ± 1.1 | 126.3 ± 1.1 | 191.9 ± 1.1 | <0.0001 |
| Thiamin (mg/1000 kcal) | 0.49 ± 0.002 | 0.49 ± 0.002 | 0.54 ± 0.002 | <0.0001 | 0.47 ± 0.002 | 0.50 ± 0.002 | 0.56 ± 0.002 | <0.0001 |
| Riboflavin (mg/1000 kcal) | 0.60 ± 0.003 | 0.64 ± 0.003 | 0.70 ± 0.003 | <0.0001 | 0.55 ± 0.003 | 0.63 ± 0.003 | 0.76 ± 0.003 | <0.0001 |
| Niacin (mg/1000 kcal) | 8.4 ± 0.04 | 9.2 ± 0.04 | 10.0 ± 0.04 | <0.0001 | 8.2 ± 0.04 | 9.0 ± 0.04 | 10.4 ± 0.04 | <0.0001 |
| Vitamin B-6 (mg/1000 kcal) | 0.60 ± 0.003 | 0.70 ± 0.002 | 0.81 ± 0.003 | <0.0001 | 0.57 ± 0.002 | 0.70 ± 0.002 | 0.85 ± 0.002 | <0.0001 |
| Vitamin B-12 (μg/1000 kcal) | 3.0 ± 0.05 | 3.7 ± 0.05 | 4.1 ± 0.05 | <0.0001 | 2.8 ± 0.05 | 3.5 ± 0.05 | 4.5 ± 0.05 | <0.0001 |
| Folate (μg/1000 kcal) | 159 ± 1.1 | 196 ± 1.0 | 227 ± 1.1 | <0.0001 | 142 ± 1.0 | 187 ± 0.9 | 252 ± 1.0 | <0.0001 |
| Pantothenic acid (mg/1000 kcal) | 2.9 ± 0.01 | 3.2 ± 0.01 | 3.5 ± 0.01 | <0.0001 | 2.8 ± 0.01 | 3.2 ± 0.01 | 3.7 ± 0.01 | <0.0001 |
| Vitamin C (mg/1000 kcal) | 51.6 ± 0.5 | 69.1 ± 0.5 | 90.2 ± 0.5 | <0.0001 | 46.9 ± 0.5 | 69.6 ± 0.5 | 94.5 ± 0.5 | <0.0001 |
| Sodium (mg/1000 kcal) | 2353 ± 10 | 2245 ± 9 | 2076 ± 10 | <0.0001 | 2499 ± 9 | 2178 ± 9 | 1998 ± 9 | <0.0001 |
| Potassium (mg/1000 kcal) | 1229 ± 5 | 1427 ± 5 | 1642 ± 5 | <0.0001 | 1139 ± 4 | 1396 ± 4 | 1763 ± 4 | <0.0001 |
| Calcium (mg/1000 kcal) | 235 ± 2 | 265 ± 1 | 309 ± 1 | <0.0001 | 213 ± 1 | 257 ± 1 | 338 ± 1 | <0.0001 |
| Magnesium (mg/1000 kcal) | 123 ± 0.5 | 143 ± 0.4 | 163 ± 0.5 | <0.0001 | 120 ± 0.4 | 139 ± 0.4 | 170 ± 0.4 | <0.0001 |
| Phosphorus (mg/1000 kcal) | 505 ± 1 | 547 ± 1 | 590 ± 1 | <0.0001 | 489 ± 1 | 538 ± 1 | 615 ± 1 | <0.0001 |
| Iron (mg/1000 kcal) | 3.8 ± 0.02 | 4.4 ± 0.02 | 4.8 ± 0.02 | <0.0001 | 3.6 ± 0.02 | 4.2 ± 0.02 | 5.2 ± 0.02 | <0.0001 |
| Zinc (mg/1000 kcal) | 4.25 ± 0.01 | 4.40 ± 0.01 | 4.44 ± 0.01 | <0.0001 | 4.10 ± 0.01 | 4.34 ± 0.01 | 4.66 ± 0.01 | <0.0001 |
| Copper (mg/1000 kcal) | 0.59 ± 0.002 | 0.65 ± 0.002 | 0.69 ± 0.002 | <0.0001 | 0.57 ± 0.002 | 0.64 ± 0.002 | 0.72 ± 0.002 | <0.0001 |
| Manganese (mg/1000 kcal) | 1.8 ± 0.01 | 2.0 ± 0.01 | 2.2 ± 0.01 | <0.0001 | 1.8 ± 0.01 | 2.0 ± 0.01 | 2.3 ± 0.01 | <0.0001 |

^1^ Values are means ± SEs. Adjustment was made for age (y, continuous), sex, weight status (underweight, normal weight, or overweight/obese), occupation (professional/manager, sales/service/clerical, security/transportation/labor, or not in paid employment), and current smoking (no or yes). For both HEI-2015 and NRF9.3, a higher total score indicates a higher diet quality. HEI, Healthy Eating Index; NRF9.3, Nutrient-Rich Food Index 9.3, RAE, retinol activity equivalent.

^2^ Calculated by using general linear models.
