# Supplementary material for: Application of the Healthy Eating Index-2015 and the Nutrient-Rich Food Index 9.3 for assessing overall diet quality in the Japanese context: Different nutritional concerns from the US
Source: PLoS One. 2020 Jan 30;15(1):e0228318. doi: 10.1371/journal.pone.0228318 (PMC6992222; doi:10.1371/journal.pone.0228318)
Supplement: S2 Table — (DOCX) [file pone.0228318.s004.docx]

S2 Table. Food group intakes (g/1000 kcal) according to tertile (T) category of total scores of HEI-2015 and NRF9.3 among participants aged ≥20 y in the Japanese National Health and Nutrition Survey 2012 (*n* = 19717)^1^

|  |  | HEI-2015 |  |  |  | NRF9.3 |  |  |
| --- | --- | --- | --- | --- | --- | --- | --- | --- |
|  | T1 (median 43.6;  *n* = 6572) | T2 (median 52.2;  *n* = 6573) | T3 (median 60.6;  *n* = 6572) | *P* for trend^2^ | T1 (median 341;  *n* = 6572) | T2 (median 452;  *n* = 6572) | T3 (median 565;  *n* = 6572) | *P* for trend^2^ |
| Rice | 186 ± 1 | 187 ± 1 | 160 ± 1 | <0.0001 | 182 ± 1 | 182 ± 1 | 168 ± 1 | <0.0001 |
| Bread | 19 ± 0.3 | 17 ± 0.3 | 15 ± 0.3 | <0.0001 | 18 ± 0.3 | 17 ± 0.3 | 16 ± 0.3 | <0.0001 |
| Noodles | 49 ± 0.8 | 31 ± 0.7 | 29 ± 0.8 | <0.0001 | 56 ± 0.7 | 31 ± 0.7 | 22 ± 0.7 | <0.0001 |
| Other grains | 7.9 ± 0.2 | 8.1 ± 0.2 | 7.8 ± 0.2 | 0.80 | 8.3 ± 0.2 | 8.0 ± 0.2 | 7.5 ± 0.2 | 0.01 |
| Potatoes | 27 ± 0.4 | 30 ± 0.4 | 31 ± 0.4 | <0.0001 | 23 ± 0.4 | 30 ± 0.4 | 35 ± 0.4 | <0.0001 |
| Sugar | 4.3 ± 0.07 | 4.4 ± 0.06 | 4.3 ± 0.07 | 0.96 | 4.5 ± 0.07 | 4.3 ± 0.06 | 4.2 ± 0.07 | 0.02 |
| Pulses | 25 ± 0.5 | 35 ± 0.5 | 44 ± 0.5 | <0.0001 | 26 ± 0.5 | 33 ± 0.5 | 46 ± 0.5 | <0.0001 |
| Nuts | 0.7 ± 0.06 | 1.1 ± 0.05 | 1.9 ± 0.06 | <0.0001 | 0.7 ± 0.05 | 1.1 ± 0.05 | 1.9 ± 0.05 | <0.0001 |
| Vegetables | 142 ± 1 | 165 ± 1 | 185 ± 1 | <0.0001 | 120 ± 1 | 160 ± 1 | 212 ± 1 | <0.0001 |
| Fruits | 23 ± 0.7 | 54 ± 0.7 | 98 ± 0.7 | <0.0001 | 36 ± 0.8 | 57 ± 0.8 | 83 ± 0.8 | <0.0001 |
| Fish | 33 ± 0.5 | 44 ± 0.4 | 48 ± 0.5 | <0.0001 | 34 ± 0.5 | 41 ± 0.4 | 49 ± 0.5 | <0.0001 |
| Meat | 45 ± 0.4 | 40 ± 0.4 | 37 ± 0.4 | <0.0001 | 44 ± 0.4 | 42 ± 0.4 | 36 ± 0.4 | <0.0001 |
| Eggs | 18 ± 0.2 | 19 ± 0.2 | 18 ± 0.2 | 0.12 | 18 ± 0.2 | 19 ± 0.2 | 18 ± 0.2 | 0.06 |
| Dairy products | 42 ± 0.8 | 47 ± 0.8 | 62 ± 0.8 | <0.0001 | 35 ± 0.8 | 49 ± 0.8 | 68 ± 0.8 | <0.0001 |
| Fats and oils | 4.9 ± 0.05 | 5.0 ± 0.05 | 5.1 ± 0.05 | 0.01 | 5.5 ± 0.05 | 5.2 ± 0.05 | 4.3 ± 0.05 | <0.0001 |
| Confectioneries | 17 ± 0.3 | 14 ± 0.3 | 12 ± 0.3 | <0.0001 | 16 ± 0.3 | 15 ± 0.3 | 13 ± 0.3 | <0.0001 |
| Fruit juice | 2.5 ± 0.3 | 3.1 ± 0.3 | 3.8 ± 0.3 | 0.0009 | 2.4 ± 0.3 | 3.0 ± 0.3 | 3.9 ± 0.3 | <0.0001 |
| Vegetable juice | 4.2 ± 0.3 | 5.1 ± 0.3 | 6.0 ± 0.3 | 0.0003 | 2.6 ± 0.3 | 5.3 ± 0.3 | 7.4 ± 0.3 | <0.0001 |
| Soft drinks | 28 ± 0.8 | 21 ± 0.8 | 20 ± 0.8 | <0.0001 | 30 ± 0.8 | 20 ± 0.8 | 18 ± 0.8 | <0.0001 |
| Tea and coffee | 278 ± 3 | 285 ± 3 | 287 ± 3 | 0.04 | 252 ± 3 | 286 ± 3 | 311 ± 3 | <0.0001 |
| Seasonings | 51 ± 0.6 | 45 ± 0.6 | 46 ± 0.6 | <0.0001 | 56 ± 0.6 | 45 ± 0.6 | 41 ± 0.6 | <0.0001 |

^1^ Values are means ± SEs. Adjustment was made for age (y, continuous), sex, weight status (underweight, normal weight, or overweight/obese), occupation (professional/manager, sales/service/clerical, security/transportation/labor, or not in paid employment), and current smoking (no or yes). For both HEI-2015 and NRF9.3, a higher total score indicates a higher diet quality. HEI, Healthy Eating Index; NRF9.3, Nutrient-Rich Food Index 9.3, RAE, retinol activity equivalent.

^2^ Calculated by using general linear models.
